# Generation of mouse-zebrafish hematopoietic tissue chimeric embryos for hematopoiesis and host-pathogen interaction studies

**DOI:** 10.1242/dmm.034876

**Published:** 2018-11-05

**Authors:** Margarita Parada-Kusz, Cristina Penaranda, Elliott J. Hagedorn, Anne Clatworthy, Anil V. Nair, Jonathan E. Henninger, Christoph Ernst, Brian Li, Raquel Riquelme, Humberto Jijon, Eduardo J. Villablanca, Leonard I. Zon, Deborah Hung, Miguel L. Allende

**Affiliations:** 1 Center for Genome Regulation, Facultad de Ciencias, Universidad de Chile, Santiago 8370415, Chile; 2 Broad Institute of MIT and Harvard, Cambridge, MA 02142, USA; 3 Department of Molecular Biology, Massachusetts General Hospital, Boston, MA 02114, USA; 4 Center for Computational and Integrative Biology, Massachusetts General Hospital, Boston, MA 02114, USA; 5 Department of Genetics, Harvard Medical School, Boston, MA 02115, USA; 6 Stem Cell Program and Division of Hematology/Oncology, Boston Children's Hospital and Dana-Farber Cancer Institute, Howard Hughes Medical Institute, Harvard Stem Cell Institute, Harvard Medical School, Boston, MA 02115, USA; 7 Center for Systems Biology, Program in Membrane Biology, Division of Nephrology, Massachusetts General Hospital and Harvard Medical School, Boston, MA 02114, USA; 8 Gastrointestinal Research Group, Faculty of Medicine, University of Calgary, Calgary, AB T2N 4Z6, Canada; 9 Immunology and Allergy, Department of Medicine, Solna, Karolinska Institute and University Hospital, Stockholm SE-171 76, Sweden; 10 Department of Cell Biology and Human Anatomy, University of California, Davis, Davis, CA 95616, USA

**Keywords:** Zebrafish, Xenotransplantation, Live imaging, Hematopoiesis, Cell migration, Host-pathogen interactions

## Abstract

Xenografts of the hematopoietic system are extremely useful as disease models and for translational research. Zebrafish xenografts have been widely used to monitor blood cancer cell dissemination and homing due to the optical clarity of embryos and larvae, which allow unrestricted *in vivo* visualization of migratory events. Here, we have developed a xenotransplantation technique that transiently generates hundreds of hematopoietic tissue chimeric embryos by transplanting murine bone marrow cells into zebrafish blastulae. In contrast to previous methods, this procedure allows mammalian cell integration into the fish developmental hematopoietic program, which results in chimeric animals containing distinct phenotypes of murine blood cells in both circulation and the hematopoietic niche. Murine cells in chimeric animals express antigens related to (i) hematopoietic stem and progenitor cells, (ii) active cell proliferation and (iii) myeloid cell lineages. We verified the utility of this method by monitoring zebrafish chimeras during development using *in vivo* non-invasive imaging to show novel murine cell behaviors, such as homing to primitive and definitive hematopoietic tissues, dynamic hematopoietic cell and hematopoietic niche interactions, and response to bacterial infection. Overall, transplantation into the zebrafish blastula provides a useful method that simplifies the generation of numerous chimeric animals and expands the range of murine cell behaviors that can be studied in zebrafish chimeras. In addition, integration of murine cells into the host hematopoietic system during development suggests highly conserved molecular mechanisms of hematopoiesis between zebrafish and mammals.

This article has an associated First Person interview with the first author of the paper.

## Abstract (precis)

**Editor's choice:** A new method to xenotransplant murine bone marrow cells into zebrafish blastulae that generates hundreds of transient chimeric animals with functional murine blood progenitor cells and innate immune cells.

## INTRODUCTION

Xenotransplantation has been a powerful tool for understanding mammalian cancer, hematopoiesis, immunity and infectious diseases. For example, humanized mice, where animals are engrafted with human cord-blood-derived hematopoietic stem cells or human adult peripheral blood mononuclear cells, allow for analysis of the cellular and molecular processes that mediate human hematopoietic and immune cell interactions within stromal niches and with pathogens *in vivo* (Ito et al., 2012; Shultz et al., 2012; Kaushansky et al., 2014; Reinisch et al., 2016). Furthermore, xenotransplants offer the unique opportunity to study the function of human-disease-associated single nucleotide polymorphisms that are non-existent or irreproducible in other species. Current research, however, is limited by the challenges of quantitatively measuring and tracking individual cell responses to these complex events (Beltman et al., 2009; Subramanian et al., 2015; Avraham et al., 2015). Observing cellular interactions in real time would allow the identification and precise evaluation of key processes between various cells and tissues that promote or restrict responses at the appropriate time and location. Intravital microscopy has been developed to perform these analyses in mouse models but lacks resolution, and often requires more invasive follow-up procedures that can interfere with normal cell behaviors. Zebrafish embryos and larvae, in contrast, are transparent, making them ideally suited to perform *in vivo* analyses in unperturbed live animals.

Strong conservation of genes and biological processes between zebrafish and mammals has made zebrafish a well-established model for basic research of the hematopoietic and innate immune systems (de Jong and Zon, 2005; Renshaw and Trede, 2012; Li et al., 2015). Xenotransplantation assays have allowed the model to be used as an inexpensive platform for assessing cancer cell behavior and to perform drug screens *in vivo* with translational applications (Zon and Peterson, 2005; Marques et al., 2009; Corkery et al., 2011; Zhang et al., 2014; Lu et al., 2015). Recently, xenotransplantation of human CD34+ cells and multiple myeloma cells into the blood stream of zebrafish embryos evidenced that human cells disseminate to the caudal hematopoietic tissue (CHT) and actively respond to the hematopoietic niche (Staal et al., 2016; Sacco et al., 2016). In a similar context, xenotransplantation of human macrophages showed that these cells can survive and acquire an activated phenotype in the zebrafish (Paul et al., 2017). Although these studies demonstrate the scientific and clinical potential of blood cell xenotransplantation in zebrafish, current methods are limited by the number of chimeras produced, the types of cells transplanted and the range of behaviors that have been observed.

Here, we develop a fast, efficient and reproducible method that generates up to 500 transient chimeric zebrafish embryos with engrafted murine hematopoietic stem and progenitor cells (HSPCs) and myeloid lineage cells. This technique is based upon injection of murine bone marrow cells into zebrafish blastulae, which leads to mammalian cell integration into the fish hematopoietic developmental program. As proof of concept, we illustrate the value of mouse-zebrafish chimeras by showing real-time visualization of many novel murine cell behaviors. During development, murine cells could be observed actively co-migrating with endogenous zebrafish cells along the primitive and definite waves of hematopoiesis. Upon the development of the vascular system, murine cells were observed to intravasate and circulate throughout the fish body. Murine cells were also shown to display interactions with vascular endothelial cells as well as the fish caudal hematopoietic tissue. Finally, murine cells were shown to respond and interact with pathogenic bacterial cells. This straightforward methodology can be scaled up to allow rapid and efficient assays for the evaluation of genetic or pharmacological interventions on mammalian cells and for discovery of novel processes related to mammalian hematopoiesis and immune cell dynamics.

## RESULTS

### Generation of mouse-zebrafish hematopoietic tissue chimeric embryos

**Fig. 1.**
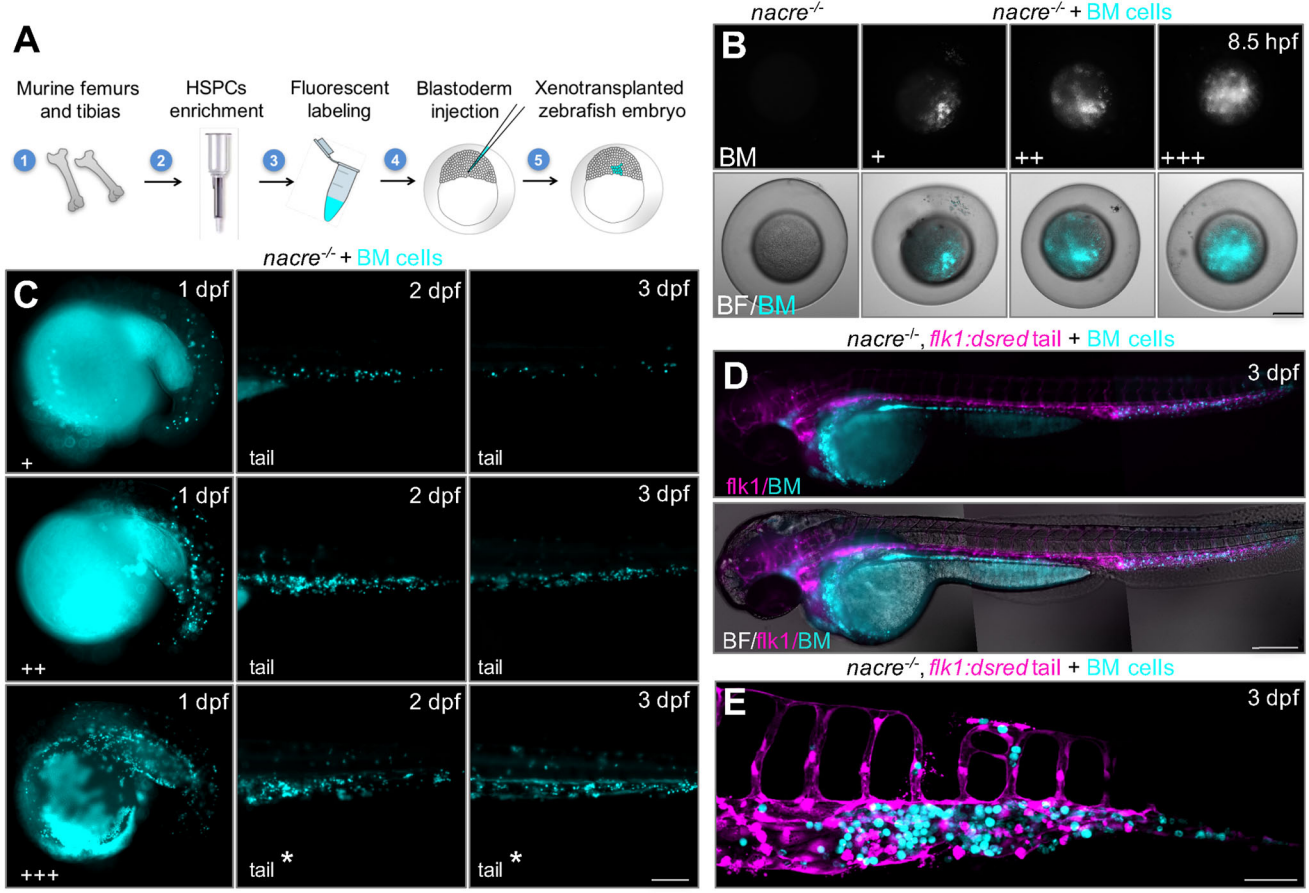
**Generation of hematopoietic tissue chimeras by transplantation of mouse bone marrow cells into zebrafish blastulae.** (A) Diagram of the experimental procedure. Mouse bone marrow cells are isolated, enriched for HSPCs by means of negative selection, blue fluorescently labeled and transplanted into the blastoderm of 3- to 5-hpf zebrafish embryos. (B) Representative epifluorescence images of the animal pole of zebrafish embryos showing 3 different levels of engraftment (+, low; ++, medium; +++, high). Scale bar: 100 µm. (C) Representative images of chimeric embryos showing mouse cells in the ICM at 1 dpf (left column), in the PBI and AGM at 2 dpf (middle column), and in the CHT at 3 dpf (right column). Asterisks indicate animals with cells circulating within the vasculature. Scale bar: 200 µm. (D) Pseudo-colored epifluorescence images of a global view of a xenotransplanted *flk1:dsred* fish at 3 dpf (mouse cells in blue; fish vasculature in magenta). Individual images from fish head, trunk and tail were joined to create a whole-embryo high-magnification image. Scale bar: 200 µm. (E) Pseudo-colored *z*-stack confocal image of the tail region of a xenotransplanted *flk1:dsred* fish at 3 dpf. Scale bar: 50 µm. BF, bright field; BM cells, mouse bone marrow cells.

The method developed here is based upon: (1) isolation of mouse bone marrow cells, (2) enrichment for HSPCs, (3) fluorescent labeling and (4) transplantation into the blastoderm of zebrafish embryos (see Materials and Methods and Supplementary Materials and Methods). First, bone marrow cells are isolated from both femurs and tibias from 1 mouse. Bone tissue is homogenized and marrow cells are collected and incubated with an antibody cocktail in order to enrich for lineage-negative cells (HSPCs) by means of negative selection. Analysis of the cell population obtained with this procedure determined that around 50% of cells are c-kit+ and 39% are double c-kit+/CD11b+ (Fig. S1). After enrichment, cells are stained for *in vivo* monitoring with a blue fluorescent emission vital dye and later injected directly into the blastoderm of zebrafish embryos at 3-5 hours post-fertilization (hpf) (Fig. 1A). To facilitate murine cell tracking and visualization at subsequent stages, *nacre^−/−^* zebrafish mutants with impaired pigmentation development were used as hosts*.* In our hands, up to 800 embryos can be transplanted in 3 h by 1 person, leading to around 500 viable embryos the following day with varying levels of engraftment (Fig. 1B). From these animals, less than 5% showed morphological abnormalities at 3 dpf.

Visualization of chimeric animals at 1 dpf evidenced transplanted cells distributing over the yolk sac and the whole body of the embryo, with 67.8±14.2% of chimeric animals showing cells within the fish intermediate cell mass (ICM) (Fig. 1C, left column). By transplanting murine cells into the transgenic endothelial vasculature reporter line *flk1:dsred*, we determined that 83.2±9.6% of chimeric animals had murine cells residing within the fish posterior blood island (PBI) and aorta-gonad-mesonephros (AGM) at 2 dpf (Fig. 1C, middle column). At 3 dpf, 93.3±9.6% had murine cells in the CHT (Fig. 1C, right column, and 1D) (mean±s.d., 2 independent experiments, *N*≥500 animals). We also observed a small fraction of chimeric animals with murine cells neighboring the developing thymic lobes at 3 dpf, suggesting that murine cells may also colonize the developing thymus, a definitive hematopoietic organ in zebrafish (Willett et al., 1997) (Fig. S2). Taken together, these results suggest active homing of murine bone marrow cells into the developing fish hematopoietic niche.

To evaluate whether trafficking to hematopoietic tissues could be visualized for other mammalian cell types transplanted into the blastoderm of zebrafish, or if it is rather a unique process for cells of the hematopoietic lineage, we xenotransplanted murine bone marrow neutrophils, murine monocyte-macrophages (LADMAC cell line), human neuroblastoma cells (SH-SY5Y cell line), human bladder epithelial cells (HTB-4 cell line) and human promyelocytic cells (HL-60 cell line). Visualization of chimeric animals shows neuroblastoma cells localizing mainly within the head region, epithelial cells localizing around the ventral anterior part of the fish body, and murine neutrophils, monocyte-macrophages and human promyelocytic cells colonizing the CHT (Figs S3-S5). These results suggest that engrafting of the zebrafish hematopoietic tissue is a specific process for cells of hematopoietic lineage, and indicate that murine and human myeloid cells can also be engrafted and followed with this procedure.

### Transplantation into zebrafish blastulas generates transient chimeric animals with variable levels of engraftment

**Fig. 2.**
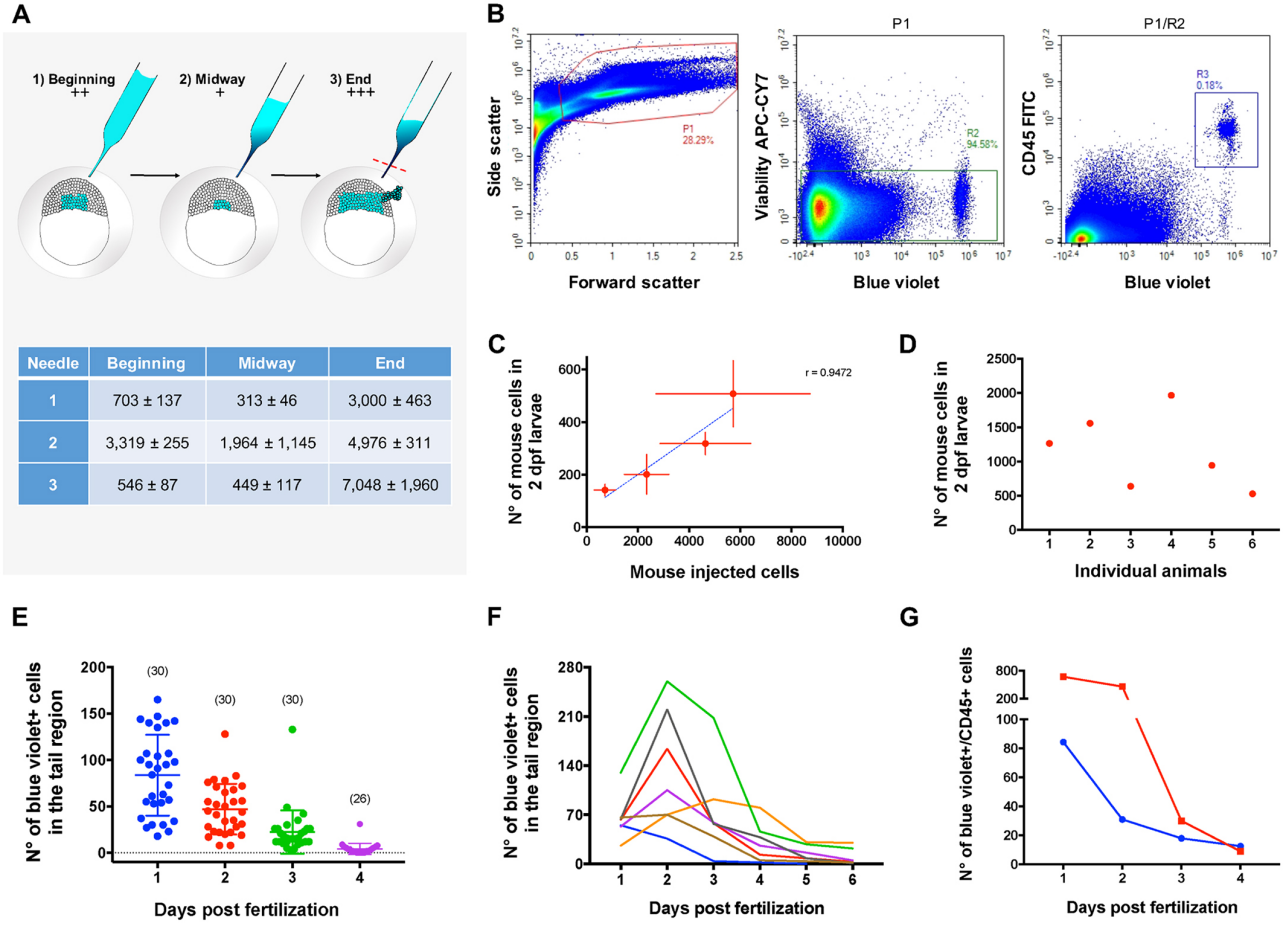
**Transplantation procedure description and quantification.** (A) Diagram of the transplantation process for a single microinjection needle (upper panel). Red dashed line represents the tip braking site. The table shows representative quantification data of mouse cells per injection for 3 different needles over time (lower panel). Data present the mean±s.d. obtained from duplicate injections. (B) Gating strategy for quantification of mouse cells in chimeric embryos. Murine cells were viable/blue violet+/CD45+ gated (R3 events, i.e., CD45+/blue violet+ cells). P1, cells; R2, viable cells. (C) Correlation between transplanted mouse cells versus engrafted mouse cells in 2-dpf embryos. Blastulae were injected with a range of ∼1000-6000 cells and the mean number of engrafted cells was determined by flow cytometry (viable/blue violet+/CD45+ gated). The mean number of mouse cells per larvae was determined from selected animals representing each of the 3 engraftment categories (+, low; ++, medium; +++, high). Mean±s.e.m. for transplantation variability (horizontal bars) and engraftment variability (vertical bars). *N*=3 replicates of 3 animals per engraftment category. Linear regression Pearson correlation *r*=0.9472, R square=0.8971, two-tailed *P*-value=0.0528. (D) Quantification of mouse cell numbers in 2-dpf individual embryos. Animals were transplanted with ∼4000 cells and individual whole-body cell suspensions were prepared for 6 selected animals from the engraftment levels ++ and +++. The number of mouse cells was determined by flow cytometry (viable/blue violet+/CD45+ gated). (E) Quantification of mouse cells in the tail region of chimeric animals by epifluorescence microscopy. Animals were transplanted with ∼2000 cells and animals representing the 3 levels of engraftment were analyzed. Numbers in parenthesis represent numbers of animals analyzed at each day post-fertilization. Results are representative of 2 independent experiments. (F) Quantification of murine cells in the tail region of 7 representative individual animals at the indicated days post-fertilization by epifluorescence microscopy. From this analysis, 12/20 animals presented a peak of colonization at 2 dpf. Results are representative of 2 independent experiments. (G) Quantification of total mouse cells in chimeric animals at the indicated days post-fertilization. Animals were transplanted with ∼2000 cells (blue line) or ∼3500 cells (red line) and whole-body cell suspensions from groups of 20 animals representing the 3 levels of engraftment were analyzed by flow cytometry (viable/blue violet+/CD45+ gated).

A quantitative analysis of the blastula transplantation procedure determined that the varying levels of engraftment observed in the chimeric animals arise from the microinjection procedure. From a single needle loaded with 5 µl of cell suspension, more than 100 embryos can be transplanted. As cells are being injected into the embryos (beginning; medium level of engraftment, ++), the cell suspension starts to concentrate at the tip of the needle, clogging the exit and reducing the number of injected cells (midway; low level of engraftment, +). The needle is then unclogged by braking the tip with tweezers, which leads to subsequent injection of the highest numbers of cells (high level of engraftment, +++). Nevertheless, some cells are expelled from the blastula and remain inside the chorion (Fig. 2A). Transplantation of varying amounts of murine cells leads to a strong correlation between the number of initially transplanted murine cells and the number of engrafted cells remaining after 2 days of development (Fig. 2B,C). Animals transplanted with 4000 cells at the blastula stage can display engraftment of up to 2000 cells by 2 dpf (Fig. 2D). These results indicate that blastula-stage zebrafish embryos can incorporate a wide range of xenografted murine hematopoietic cells.

We next evaluated murine cell numbers in the tail region of chimeric animals over time, focusing on the caudal area where hematopoiesis occurs in the zebrafish. Visualization of mouse cells by epifluorescence microscopy showed a continuous reduction of engrafted cells during fish development. Imaging of chimeric animals transplanted with 2000 cells showed animals displaying on average around 80 murine cells in the tail region at 1 dpf, 50 cells at 2 dpf, 25 cells at 3 dpf and 5 cells at 4 dpf (Fig. 2E). We then evaluated and followed individual chimeric animals transplanted with ∼3500 cells. Imaging of murine cell numbers in the tail region showed that around half of the analyzed animals have a peak of colonization of the hematopoietic tissue at 2 dpf that later declines towards 6 dpf (Fig. 2F, and Figs S6 and S7). These results suggest a wave of murine cell engraftment of the hematopoietic tissue that peaks at 2 dpf, to later decline with time.

To address whether viable murine cells could still be detected in the chimeras regardless of loss of the vital dye detection by epifluorescence imaging, we analyzed chimeras for the presence of murine cells by flow cytometry. Evaluation of viable murine CD45+ cells at different days post-fertilization determined that the numbers of CD45+ murine cells detectable by flow cytometry decreased steadily over time (Fig. 2G and Fig. S8), indicating that disappearance of these cells post-engraftment is not simply due to dilution of the vital dye. Rather, these results demonstrate that transplantation of bone marrow cells in the blastula stage of zebrafish embryos generates transient chimeric animals.

### Xenotransplantation of murine cells into zebrafish blastulae does not induce vital dye transfer or cell fusion events

**Fig. 3.**
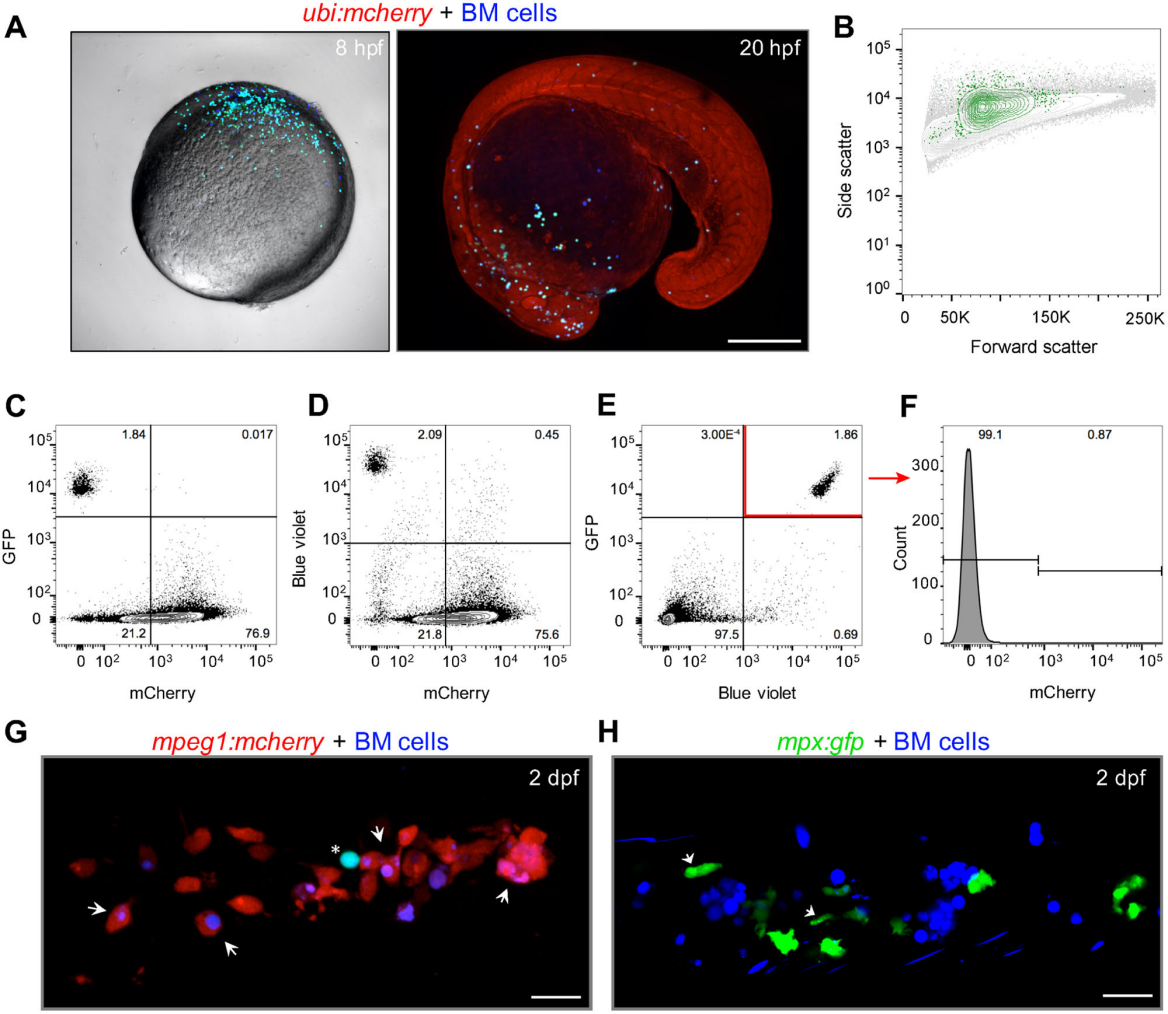
**Transplantation of mouse cells into zebrafish blastulae does not result in cell fusion events or vital dye transfer.** (A) Mouse bone marrow cells (UBI-GFP) were blue-labeled and transplanted into *ubi:mcherry* transgenic zebrafish blastulae. Confocal images from an 8-hpf (left panel) and a 20-hpf (right panel) transplanted embryo show no triple-labeled cells (red, green and blue; white emission). Scale bar: 250 µm. (B) At 2 dpf, 12 transplanted embryos were selected and a whole-embryo cell suspension was prepared and analyzed by flow cytometry. Contour plot of the physical parameters identified by forward and side scatter show back-gated mouse bone marrow cells (green events) and fish cells (gray events). (C-E) Contour plots from color-based analysis show (C) 0.017% of events as GFP+ and mCherry+, (D) 0.45% of events as blue violet+ cells and mCherry+, and (E) the majority of GFP+ events as blue violet+. (F) mCherry+ histogram plot of blue violet+/GFP+ pre-gated events (red arrow in E) showing 0.87% of triple-labeled cells. (G) Mouse bone marrow cells (UBI-GFP) were blue-labeled and transplanted into *ubi:mcherry* transgenic zebrafish blastulae. Confocal *z*-stack image of the fish PBI showing macrophage (red labeled) internalization of mouse blue+/GFP− cells (white arrows). Asterisk indicates a double blue- and green-labeled cell (cyan). Scale bar: 20 μm. (H) Confocal pseudo-colored *z*-stack image of the fish PBI from an *mpx:gfp* fish transplanted with blue-labeled mouse bone marrow cells showing activated neutrophil cells (green labeled, white arrows) around mouse cells. Scale bar: 20 μm. BM cells: mouse bone marrow cells.

To exclude the possibility that the vital dye used to stain the murine cells could be transferring to fish cells, or that murine cells may be fusing with fish cells after xenotransplantation, we transplanted blue-stained bone marrow cells from UBI-GFP transgenic (ubiquitously green) mice into *ubi:mcherry* transgenic fish. These chimeras have green- and blue-labeled murine cells in a ubiquitously red fluorescent zebrafish host. Confocal imaging of an 8-hpf and a 20-hpf transplanted animal showed no triple-color labeled cells (white emission), suggesting negligible dye transfer or cell fusion events (Fig. 3A). To corroborate these results, cells from 2 dpf chimeric animals were analyzed by flow cytometry. Examination of the physical parameters of live pre-gated cells showed murine bone marrow cells as a uniform population that are, on average, slightly larger than fish cells (Fig. 3B). Cell-color emission analysis detected scarce cells showing double green and red label (0.017%), as well as few cells double labeled blue and red (0.45%) (Fig. 3C,D). In addition, analysis of green- and blue-labeled cells demonstrated that all green cells are blue, indicating that all transplanted cells retain the blue stain during this timeframe (Fig. 3E). Finally, to test for the existence of triple-colored cells, all green- and blue-labeled cells were tested for red emission, showing that only a small fraction (0.87%) are triple labeled (Fig. 3F). These results indicate that transplantation of stained murine cells does not lead to significant vital dye transfer to fish cells and that cell fusion events are rare.

Nonetheless, we wished to evaluate the origin of double- and triple-labeled cells. To ascertain whether endogenous cell-phagocytosis of murine cells could explain these events, we analyzed innate immune cell responses in the chimeras. First, we transplanted blue-stained bone marrow cells from the UBI-GFP transgenic mice into the macrophage-labeled *mpeg1:mcherry* transgenic reporter fish. We found that endogenous hematopoiesis is not affected despite the presence of mammalian cells. However, live imaging showed that some fish macrophages residing in the CHT contained blue, but not green, labeled cell material (Fig. 3G). This suggests that endogenous immune cells may recognize and phagocytose murine cell debris or dying murine cells that have lost GFP expression. We next transplanted blue-stained bone marrow cells into the neutrophil-labeled *mpx:gfp* transgenic reporter fish. As before, neutrophil development proceeded normally. A fraction of the fish neutrophils displayed an activated morphology in the chimeras, but showed no internalization of murine cells (∼100 larvae) (Fig. 3H). These results suggest that macrophages, but not neutrophils, phagocytose dying murine engrafted cells.

We next evaluated the extent to which murine xenotransplanted cells underwent cell death. We analyzed apoptotic cell death markers in freshly isolated murine bone marrow cells prior to enrichment, HSPC-enriched and stained bone marrow cells prior to transplantation, and xenotransplanted bone marrow cells in 2-dpf chimeric animals. Freshly isolated bone marrow cells showed ∼13% dying cells, whereas enriched and stained bone marrow cells showed ∼16% dying cells (Fig. S9A,B). Analysis of apoptosis markers in the chimeras at 2 dpf showed that 5.4% of xenografted cells were TUNEL positive (Fig. S9C,D). This result suggests that there are few dying murine cells in the chimeras at 2 dpf, which is in agreement with the flow cytometry analyses that detected less than 1% double- or triple-labeled cells. Overall, these results indicate that, with this procedure, a high number of murine cells can be transplanted into zebrafish blastulae that remain viable during the early larval stage. It also suggests that endogenous fish macrophages are eliminating engrafted cells.

### Chimeric animals have murine hematopoietic progenitors and myeloid lineage cells

**Fig. 4.**
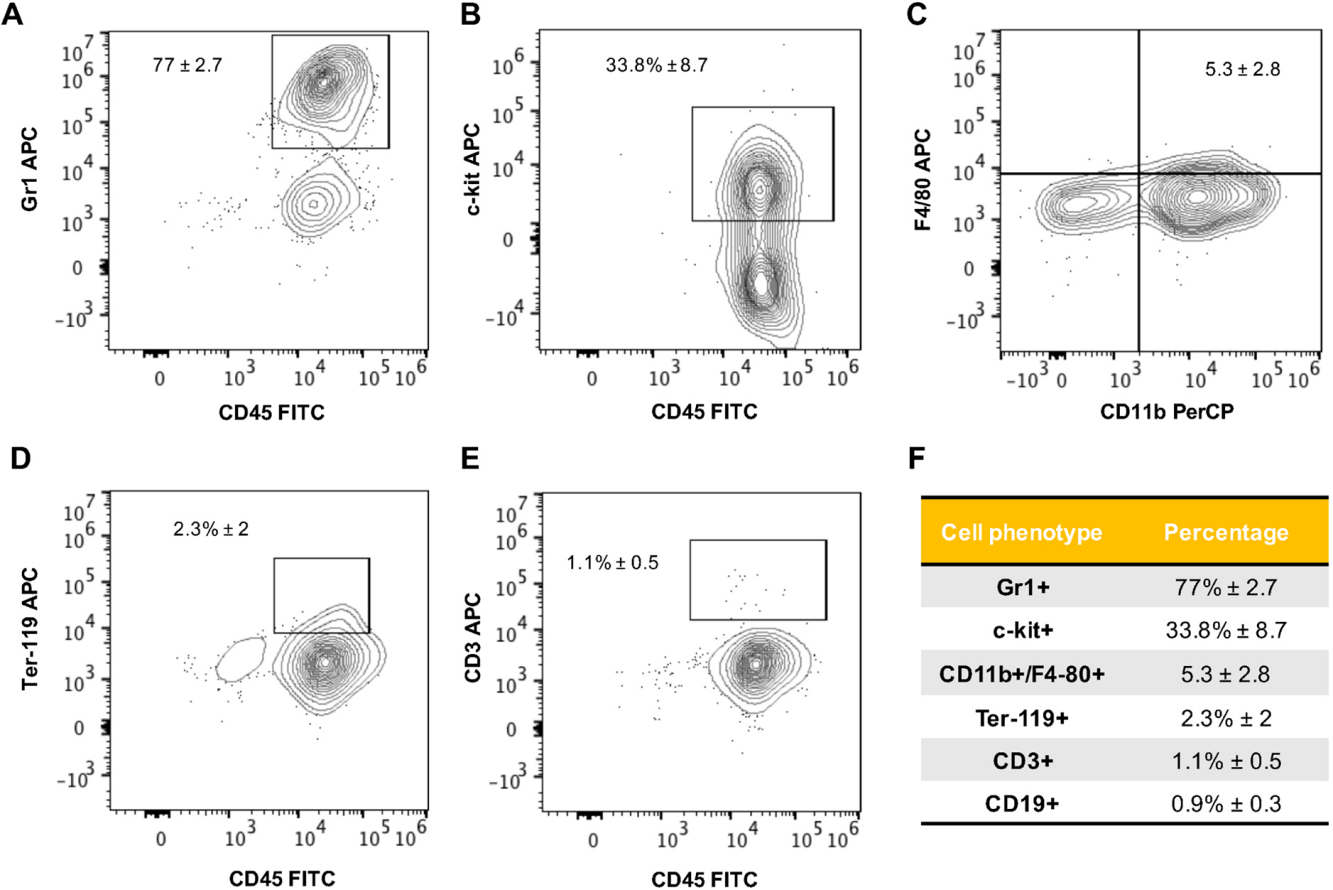
**Engrafted murine cells in chimeric animals include hematopoietic progenitors and myeloid lineage cells.** Mouse bone marrow cells were blue-labeled and transplanted into zebrafish blastulae. At 2 dpf, 100 chimeric animals were selected, and a whole-embryo cell suspension was prepared and analyzed by flow cytometry. Representative contour plots from color-based analysis show (A) 77% of viable/blue violet+/CD45+ events as Gr1+, (B) 33% as c-kit+, (C) 5% of events as CD11b/F4-80+, (D) 2% of events as Ter-119+ and (E) less than 1% of events as CD3+/CD19+. (F) Table summarizing mouse cell phenotypes in chimeric animals at 2 dpf. Results are representative from 2 independent experiments consisting of 3 biological replicates.

We next evaluated whether we could distinguish different cell differentiation markers in the murine cell population present in the chimeric animals. Flow cytometry analysis of whole-body cell suspensions from 2 dpf animals determined that around 77% of murine cells expressed the granulocyte-cell-associated Gr1 protein (Fig. 4A) and 33% expressed the c-kit protein (Fig. 4B), which is highly expressed in hematopoietic stem and progenitor cells. In addition, 5% expressed the bone-marrow-derived monocyte-macrophage CD11b/F4/80 proteins (Fig. 4C) and 2% expressed the Ter119 protein associated with erythrocytes (Fig. 4D). Evaluation of B- and T-cell differentiation markers determined that less than 1% of murine cells expressed the CD19 and CD3 proteins, respectively (Fig. 4E,F). These results suggest that chimeric animals have murine progenitor as well as differentiated cells that mostly belong to the myeloid lineage.

We then evaluated murine cell differentiation markers present specifically in the fish caudal hematopoietic tissue by fluorescent imaging and whole-mount immunofluorescence. Confocal imaging of the CHT of chimeric animals showed that murine bone marrow cells have similar patterns of intracellular granules and morphology to fish cells in the CHT (Fig. S10). Immunofluorescence assays performed in chimeric embryos showed that 35.6% of murine cells express the c-kit protein and that 21.1% of cells express the ki-67 protein related to active cell proliferation (Figs S11 and S12). A few chimeric embryos displayed morphological abnormalities. Among these, we observed animals with an enlarged CHT containing varying levels of blue fluorescence emission (Fig. S13). Since the vital dye utilized to track murine cells in our experiments becomes diluted between mother and daughter cells upon cell division, the observed heterogeneous levels of fluorescence suggest that unregulated or excessive local murine cell proliferation can lead to deformation of the host niche. We next evaluated myeloid cell lineage markers. Our analyses revealed that 32.5% of murine cells in the hematopoietic niche expressed the Gr1 protein and 13.4% of cells expressed the F4/80 protein (Figs S14 and S15). Altogether, these results suggest that chimeric animals contain different murine cell phenotypes consisting of proliferating murine hematopoietic progenitors as well as myeloid lineage cells.

### Real-time tracking of murine bone marrow cells in zebrafish reveals heterogeneous cell behaviors

**Fig. 5.**
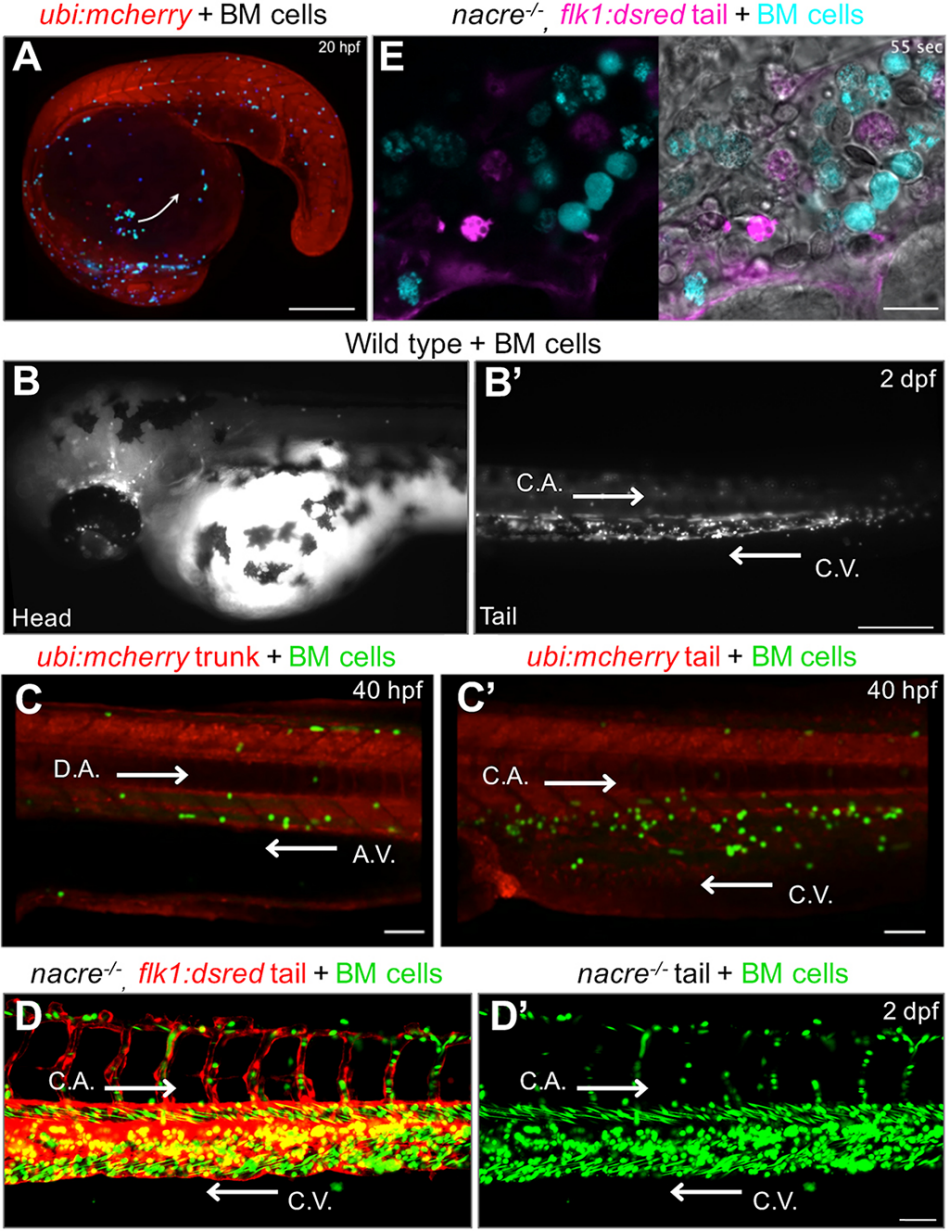
**Live imaging of chimeric zebrafish embryos and larvae shows migration and behavior of mouse bone marrow cells.** (A) Single-slice confocal image obtained from a 13-h time-lapse sequence that shows mouse bone marrow (BM) cells migrating along the route of endogenous primitive macrophages. Arrow depicts cell migration direction over the yolk sac (see Movie 1). Scale bar: 200 μm. (B,B′) Epifluorescence images obtained from a time-lapse sequence that show white-colored mouse cells circulating within the (B) fish head and (B′) tail vasculature (see Movie 2). Scale bar: 250 μm. (C,C′) Single-slice confocal images from a time-lapse sequence showing UBI-GFP transgenic mouse cells (green) interacting with fish endothelial cells within (C) the fish trunk dorsal aorta and axial vein, and (C′) the tail caudal aorta and caudal vein in an *ubi:mcherry* fish (see Movies 3 and 4). Scale bars: 50 μm. (D,D′) Pseudo-colored confocal images captured from a 1.5-h time-lapse sequence showing green mouse cell dynamics within the fish caudal hematopoietic tissue in a vasculature reporter *flk1:dsred* animal. Panel D′ is the green channel of panel D (see Movie 5). Scale bar: 50 μm. (E) Single-slice pseudo-colored confocal image from a time-lapse sequence that shows individual mouse cell (cyan) dynamics at high magnification inside the fish CHT in a *flk1:dsred* animal (see Movie 6). Scale bar: 10 μm. C.A., caudal aorta; C.V., caudal vein; D.A., dorsal aorta; A.V., axial vein. White arrows in B′-D′ depict fish blood flow direction.

Using live imaging, we next analyzed the behavior of the xenotransplanted murine bone marrow cells during larval development. Chimeric animals at around 20 hpf showed murine cells distributed and moving over the yolk sac along the migratory route of endogenous primitive macrophages (Herbomel et al., 1999) (Fig. 5A and Movie 1). Visualization of animals at 1 dpf showed that a subset of murine cells had entered the fish circulatory system, a phenomenon observed regardless of the total xenografted cell number. At 2 dpf, murine cells could be observed circulating within the entire fish bloodstream (Fig. 5B and Movie 2). Visualization at higher magnification revealed that circulating murine cells displayed variable velocities and adhesive rolling behaviors, indicative of differential adherence to the vascular endothelium (Fig. 5C,C′, and Movies 3 and 4). Similarly, visualization of murine and fish cells within the CHT showed highly motile behaviors, which suggest dynamic niche interactions (Fig. 5D,E, and Movies 5 and 6). Overall, these results show that transplantation of murine cells at the blastula stage allows the visualization of novel murine cell behaviors and evidence that xenografted murine cells have heterogeneous phenotypes in the zebrafish chimeras.

### Xenotransplanted murine bone marrow cells respond to bacterial infection in fish

**Fig. 6.**
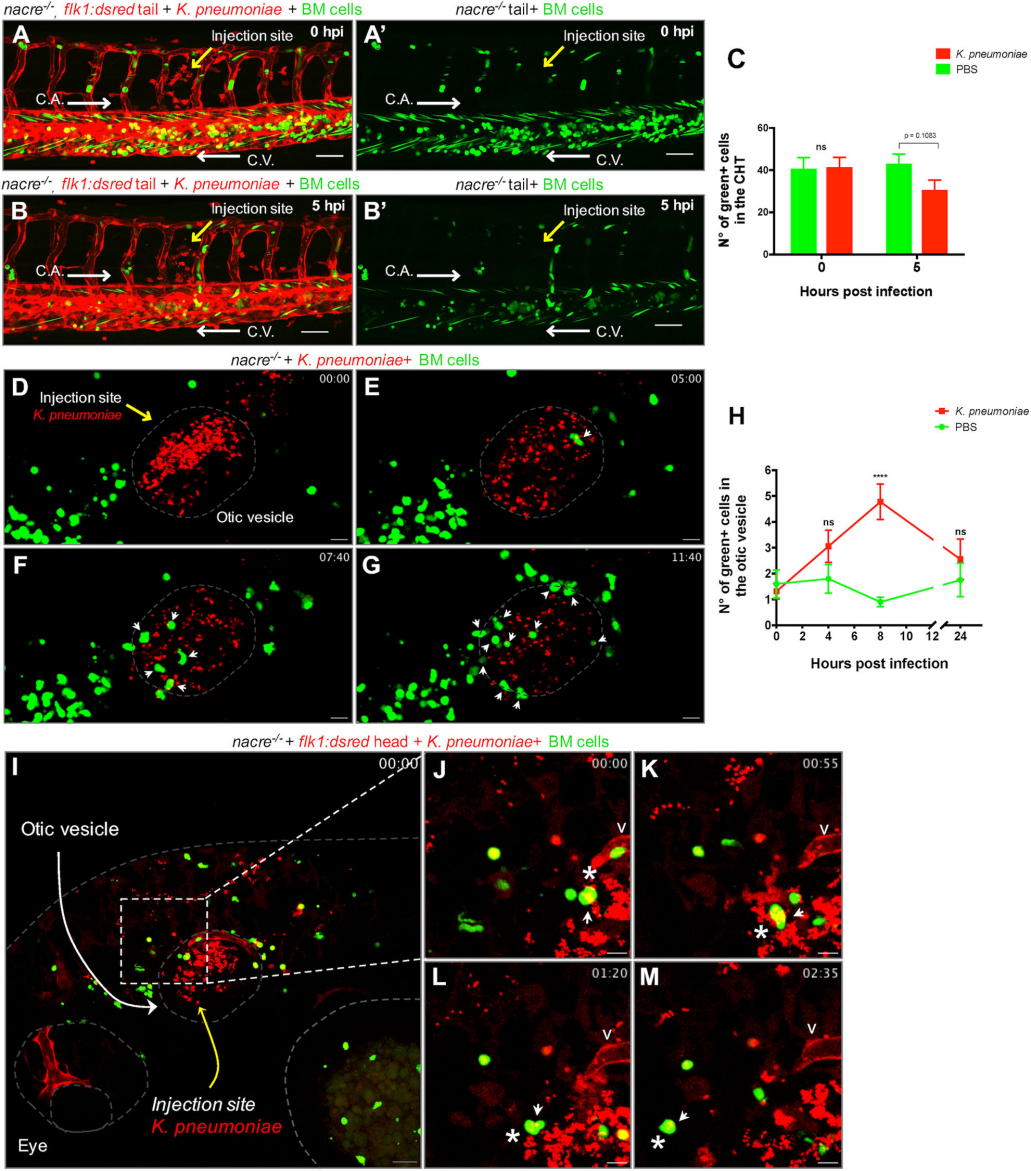
**Transplanted mouse cells respond to a bacterial infection in zebrafish.** Pseudo-colored confocal images from a time-lapse sequence of xenotransplanted *flk1:dsred* fish infected with *K. pneumoniae*. Endothelial cells are red-labeled, mouse cells are green-labeled and bacteria are red-labeled. (A-B′) Images acquired right after (A,A′; 0 hpi) or 5 h after (B,B′; 5 hpi) intramuscular infection with ∼100 cfu of *K. pneumoniae*. (A′,B′) Green channel reveals depletion of mouse cells in the CHT at 5 hpi (see Movie 7). Scale bars: 50 μm. (C) Quantification of the CHT-resident mouse cells before and after 5 h of a tail-muscle infection with *K. pneumoniae. N*=10 animals per condition. Statistical significance was analyzed by a two-tailed unpaired Student's *t*-test. (D-G) Confocal images captured from a time-lapse sequence centered on the otic vesicle of a fish infected with ∼500 cfu of *K. pneumoniae*. White arrows indicate infiltrating murine cells (see Movie 8). (H) Quantification of the otic-vesicle-resident mouse cells at the indicated time points after an infection with ∼500 cfu of *K. pneumoniae. N*=10 animals per condition. Statistical significance was analyzed by a two-way ANOVA with Bonferroni post-test (*P*≤0.001). (I) Confocal image captured from a high-resolution time-lapse sequence centered on the otic vesicle of a fish infected with ∼500 cfu of *K. pneumoniae.* Scale bar: 20 μm. (J-M) Enlarged images showing the interaction of a single mouse cell (asterisk) with bacteria (arrow) that was tracked during the indicated times; the mouse cell containing bacteria later migrates away from the infection site (see Movie 9). Scale bars: 10 μm. C.A., caudal aorta; C.V., caudal vein; v, otic vesicle vasculature; cfu, colony-forming units; hpi, hours post-infection. White arrows in A-B′ depict fish blood flow direction. Yellow arrows in images depict the bacterial microinjection site.

We had observed that murine bone-marrow-derived cells xenografted into zebrafish can express neutrophil and macrophage terminal-differentiation markers. In order to determine whether these cells are able to functionally respond to an inflammatory signal, 3-dpf chimeric larvae were infected with a clinical isolate of *Klebsiella* *pneumoniae*, an important human bacterial pathogen (Munoz-Price et al., 2013). *In vivo* visualization of the CHT of a chimeric animal after an intramuscular injection with ∼100 cfu of *K. pneumoniae* showed changes in the distribution of murine cells within the CHT compared to non-injected fish. Five hours after bacterial infection, murine cells became depleted from the hematopoietic niche (Fig. 6A-B′ and Movie 7). Intramuscular injection with a more concentrated inoculum of bacteria (∼400 cfu) showed a reduction in CHT murine cell numbers after 5 h post-infection (hpi) (Fig. 6C) and showed murine cells accumulating around the infection site with increased numbers at 24 hpi (Fig. S16). These results suggest that murine cells are able to respond to a bacterial infection. To evaluate whether murine cells are also able to respond to a sterile inflammatory signal induced by injury, 2-dpf chimeric animals were subjected to a tail-cut assay. Quantification of murine cells infiltrating the wounded area over time showed no response of murine cells (Fig. S17). These results suggest that murine cells in chimeric animals are able to respond to pathogen-associated molecular patterns (PAMPs) but not to damage-associated molecular pattern molecules (DAMPs).

To exclude the possibility that the observed murine cells responding to infection are endogenous macrophages that have phagocytosed murine cells, we transplanted blue-labeled murine bone marrow cells into the macrophage-labeled *mpeg1:mcherry* transgenic reporter fish and visualized both cells’ response to a localized tail-muscle infection. Imaging at the infection site shows murine cells responding to the infection independently to endogenous macrophages (Fig. S18). In sum, these experiments reveal that murine cells change their distribution in zebrafish chimeras upon bacterial infection and suggest they are mounting a specific response to this stimulus.

To *in vivo* visualize the interaction dynamics between murine cells and bacteria, we injected ∼500 cfu of live red-fluorescent *K. pneumoniae* into the otic vesicle of 2-dpf chimeric animals. Live imaging of the infected area showed murine cells actively interacting with bacterial cells (Fig. 6D-G and Movie 8). Quantification of infiltrated murine cells over time shows a peak of infiltration at 8 hpi (Fig. 6H). To visualize in greater detail murine cell interactions with bacterial cells, we xenotransplanted into *flk1:dsred* larvae and performed high-resolution time-lapse imaging starting at 9 hpi. Live imaging showed murine cells migrating and interacting with bacteria, with some murine cells showing an intracellular bacterial load (Fig. 6I-M and Movie 9). These results indicate that murine cells are able to detect and interact with bacterial cells in a zebrafish.

## DISCUSSION

The transparency and tolerance to experimental manipulation of zebrafish embryos has extended their use in unanticipated ways, which include the transplantation of animal, plant and bacterial cells (Toh et al., 2013; Lu et al.; 2015; Alvarez et al., 2015). For instance, a wide body of work has shown that xenotransplantation of human cancer cells into zebrafish is a useful *in vivo* platform to assess and screen for molecules controlling cancer cell metastasis and migration. Until now though, zebrafish xenotransplantation assays to visualize and study normal mammalian hematopoietic or immune cell development and function in living fish embryos have not been established. To broaden the scope of available cell xenotransplantation assays in zebrafish, we have developed a method that generates zebrafish embryos with transiently chimeric hematopoietic tissue. This procedure constitutes a simple and efficient technique that allows the engraftment of mammalian hematopoietic cells into the fish embryonic developmental hematopoietic program. As a result, mammalian cells integrate into the developing primitive and definitive hematopoietic tissues of the host, and chimeric animals grow with a heterogeneous population of mammalian bone-marrow-derived cells amidst their own hematopoietic niche. The xenografted animals allow for a broad spectrum of *in vivo* experiments to be carried out readily without any further manipulations and to continually visualize the cell behaviors that might ensue.

The method developed here to generate mammalian-zebrafish chimeras differs from previously existing ones in 4 main aspects. First, this method increases by at least 4 times the number of cells that can be xenotransplanted in zebrafish. Xenotransplantation into the fish bloodstream has been previously reported for a range of 50-500 cells (Staal et al., 2016; Sacco et al., 2016). In contrast, injection into the animal pole of a blastula shows that at least 2000 mammalian cells can be engrafted into a fish. Strikingly, the animals develop normally considering the substantial number of exogenous cells. Of note, this might be possible only for hematopoietic cells, since transplantation of similar numbers of other cell types (or transformed cells) results in developmental abnormalities and high lethality, possibly due to unregulated expression of morphogens or adhesion molecules that impair fish development (i.e. neurons and epithelial cells in Fig. S4). Second, this method broadens the range of potential experiments that can be performed in chimeric animals. Xenotransplantation of cells within the fish bloodstream leads to their localization within the vasculature of the hematopoietic niche. In contrast, xenotransplantation in the blastula stage allows cells to be integrated into the bloodstream as well as the developing hematopoietic tissues. This may allow analysis of hematopoietic cell-niche interactions. It also allows us to examine the process of homing that progenitors undergo to migrate specifically to the hematopoietic tissue. In fact, we noted that xenotransplanted cells followed the same path taken by endogenous hematopoietic progenitors, suggesting they are following the same guidance cues at the appropriate developmental time. Thirdly, it allows visualization of innate immune cell function *in vivo*. Murine innate immune cells have been shown to respond and actively interact with bacterial cells in a zebrafish for the first time, to the best of our knowledge, using this model. Fourth, this method expands the developmental time frame of experiments that can be conducted. Transplantation into the bloodstream typically occurs in 2-dpf animals, restricting experiments to later developmental stages. Transplantation into blastula-stage embryos allows experiments to be started as soon as 6 hpf. In sum, this methodology broadens zebrafish blood-cell xenotransplantation studies.

Current limitations of this method include the restricted survival of mouse cells in chimeric animals. Mouse cells could be autonomously dying because of a lack of a suitable environment needed for a long-term engraftment (e.g. lower temperature), and/or mouse cells could be actively being eliminated by endogenous macrophages. It would be interesting to evaluate whether a milder bone marrow cell isolation protocol (e.g. bone flushing) could improve the survival of mouse cells in the chimeras. Of note, the high variability of cell transplantation at the blastula stage implies that thousands of murine cells must be transplanted in order to have, on average, hundreds of cells engrafted in chimeric animals by 2 dpf. In addition, higher numbers of transplanted cells yield higher numbers of total engrafted cells, higher colonization of the CHT and more prolonged survival of murine cells in the chimeras (Fig. 2). For experiments tracking individual cell responses during early embryogenesis, a low level of engraftment (a few hundred cells) would be sufficient. In contrast, for experiments starting at later time points (after 2-3 dpf), or for infection experiments, animals with a higher level of engraftment would be necessary (thousands of cells). The partial conservation between immune system molecules in zebrafish and mammals should also be considered as a potential limitation for these experiments. Finally, we foresee that this method could be improved by utilizing immunodeficient animals, as has been shown in humanized mice (Ito et al., 2012).

Monitoring of zebrafish chimeras throughout embryonic development evidenced murine cells migrating along and colocalizing, in succession, to the fish yolk sac, ICM, AGM, PBI and, later, the CHT. In around half of the population of chimeric animals, mouse cells display a peak of colonization of the hematopoietic tissue at 2 dpf. In a few instances, we also observed xenografted cells neighboring the developing thymic lobes. These results strongly suggest that murine cells are actively sensing and responding to molecular cues originating from both the host primitive and definitive hematopoietic niches. The low frequency of chimeric animals displaying murine cells localized to the thymic lobes suggests that, perhaps, specific bone marrow cell subsets or derivatives can colonize the thymus. These cells may be poorly represented in the cell populations recovered using our selection method and their identity needs to be further determined. Since endogenous HSPCs start colonizing the developing thymus and kidney marrow at 3 dpf in order to give rise to the definitive wave of hematopoiesis in zebrafish (Murayama et al., 2006; Paik and Zon, 2010), it would be interesting to evaluate whether xenotransplanted cells localizing to the thymus also correspond to murine hematopoietic stem cells. However, it would also have to be ruled out that the presence of these cells around the developing thymic lobes might be due to chemokines emanating from the developing brain or other structures in the anterior part of the embryo's body; further work is required to explore these hypotheses. We also observed zebrafish chimeric animals that displayed murine cells within the pronephric tubules and kidney rudiment at 3 dpf (Fig. 1D). Previous reports have described a migration pathway along the pronephric tubules that initiates adult hematopoiesis in the developing kidney (Bertrand et al., 2008). However, in the chimeras, we cannot exclude that the label we observe in the kidney could be associated with elimination of murine-cell debris. Importantly, transplantation of murine neutrophils, monocyte-macrophages and human promyelocytic cells, but not human neuroblastoma and human epithelial cells, resulted in colonization of the CHT, suggesting cell lineage homing specificity. Altogether, these results demonstrate that mammalian hematopoietic cells can recapitulate early zebrafish hematopoiesis, implying high conservation of homing molecules during hematopoiesis among vertebrates. In addition, we demonstrate that zebrafish hematopoietic tissue chimeric embryos can be used to study *in vivo* and in real-time mammalian developmental hematopoietic trafficking events.

An evaluation of murine cell phenotypes in the chimeras at 2 dpf evidenced that murine cells express antigens related to stem and progenitor cells, cell proliferation, and myeloid lineage cells. Few cells expressed antigens related to erythrocytes and an even smaller percentage of cells expressed antigens related to T and B cells. The proportion of c-kit+ cells in the chimeras (∼33-35%) is slightly lower than the ∼50% of c-kit+ cells observed in the HSPC-enriched bone marrow cell population prior to transplantation. In addition, murine cells display antigens related to myeloid lineage cells after arriving at the CHT. Further, when observing live xenotransplanted larvae, we saw cells in circulation, some of which displayed ‘rolling’ behaviors, akin to what has been described for leukocyte-endothelium interactions. We hypothesize that proliferating murine hematopoietic progenitors differentiate into myeloid lineage cells in zebrafish, as has previously been reported for human CD34+ cells (Staal et al., 2016). To what extent murine cell differentiation takes place in a zebrafish, and if this is a cell-autonomous process or host driven, needs to be further addressed. What other cell types are present or become engrafted in the chimeras needs to be further explored as well. Regardless, our results reveal the existence of different cell types in the chimeras, supporting the concept that zebrafish hematopoietic tissue chimeric animals can be used to study the cell biology of mammalian HSPCs and derived cells *in vivo*, as well as their interactions within the hematopoietic niche and endothelial vasculature.

The zebrafish is an excellent model for the study of innate immunity and of the inflammatory response elicited by tissue damage or infection (Trede et al., 2004; Torraca et al., 2014; Robertson et al., 2014). Given that we had shown that murine HSPCs differentiate into neutrophils and macrophages in the fish, we wished to know whether they had acquired functional competence as well. Chimeric larvae challenged by localized intramuscular infection with *K. pneumoniae* revealed a dramatic decrease of murine cell numbers in the fish CHT, with an increase in murine cell numbers in the vessel directly proximal to the inoculum injection site. This suggests that murine cells respond to the infectious challenge by entering the circulation and homing to the bacterial site. However, we cannot exclude the possibility that part of the observed effect is due to activation of endogenous immune cells or emergency granulopoiesis that might lead to elimination of the murine cells in the CHT. Infection with a more concentrated inoculum of bacteria (∼400 cfu) resulted in specific localization of murine cells near the injection site with increased numbers of infiltrated cells over time. These results suggest that murine cells actively migrate towards the infection site, although bacterial-induced tissue necrosis might also favor the extravasation or interstitial migration of murine cells towards the infected site. In addition, murine cells can be observed actively interacting with bacterial cells when these have been injected into the otic vesicle, with some murine cells evidencing an intracellular bacterial load indicative of phagocytosis. It remains to be determined what murine immune cell types are manifesting these responses, as well to characterize which molecules they are expressing when stimulated by bacterial cells in a zebrafish. Interestingly, murine cells were not seen to home to the wounded tissue in a tail-cut assay. These results suggest that murine cells are not responding to fish-derived DAMPs. This could indicate that, in this context, DAMPs do not provide a strong enough signal to recruit the murine cells. Alternatively, they could suggest a lack of conservation of pro-inflammatory molecules that promote wound healing inflammation between zebrafish and mice. Nevertheless, our results show that xenografted murine cells are able to respond to an infection and we demonstrate, for the first time, that zebrafish chimeric animals can be utilized to study mammalian immune cell host-pathogen interactions *in vivo*.

In conclusion, our results demonstrate that murine bone marrow cell xenotransplantation in the blastula stage of zebrafish embryos expands the range of murine cell behaviors that can be studied in zebrafish chimeras. We have verified the utility of this system by showing non-invasive real-time visualization of murine bone marrow cell trafficking, and their interaction within stromal niches and pathogens *in vivo*. Both murine and human bone-marrow-derived cells can be engrafted into the fish CHT. Therefore, the range of potentially valuable applications of this simple yet powerful technique can include diverse cell types and species. Current limitations include the restricted survival of murine cells in the chimeras to up to 6 dpf. Future improvements could incorporate the use of immunodeficient zebrafish hosts to extend the survival of the xenografted cells as to benefit the analysis of mammalian cell responses without the interference of competing endogenous cells. We envision that zebrafish hematopoietic tissue chimeric embryos could be adapted to study *in vivo* and in real time the contribution of cell-autonomous versus non-cell-autonomous gene functions in diverse processes such as homing mechanisms, tolerization and host-pathogen interactions, among others. Ultimately, the efficiency of transplantation achieved with this technique could allow targeted drug screens or even patient-specific assays to be carried out rapidly in an *in vivo* experimental setting (Konantz et al., 2012; Fior et al., 2017).

## MATERIALS AND METHODS

### Animal use and generation of mouse-zebrafish chimeras

Wild-type AB, *nacre^−/−^* (Lister et al., 1999) mutants and Tg(*–3.5ubi:mCherry*), herein *ubi:mcherry* (Mosimann et al., 2011), Tg(*kdrl(flk1):dsRed2*), herein *flk1:dsred* (Kikuchi et al., 2011), Tg(*mpeg1:mCherry*) (gl22Tg), herein *mpeg1:mcherry* (Ellett et al., 2011), and Tg(*mpx:EGFP*)^i114^, herein *mpx:gfp* (Renshaw et al., 2006) transgenic embryos were raised and staged according to Westerfield (2000). Zebrafish studies were approved by the Massachusetts General Hospital Institutional Animal Care and Use Committee.

### Isolation of mouse bone marrow HSPCs and neutrophils

Mouse bone marrow cells were isolated from 6- to 8-week-old female/male C57BL/6 or CByJ.B6-Tg(UBC-GFP)30Scha/J (UBI-GFP) animals. For enrichment of HSPCs or neutrophils, bone marrow cell suspensions were incubated with lineage cell depletion kit or neutrophil isolation kit from Miltenyi Biotec (Auburn, CA). The procedure was conducted according to the manufacturer's recommendations, with 1 modification involving addition of an extra 10 µl of anti-TER-119 (Ebioscience, #13-5921-81) to the antibody cocktail.

### Murine and human cell lines

The urinary bladder epithelial T24 cell line (ATCC^®^ HTB-4™), the neuroblastoma cell line SH-SY5Y (ATCC^®^ CRL-2266™), the promyelocytic HL-60 cell line (ATCC^®^ CCL-240™) and the LADMAC bone marrow cell line (ATCC^®^ CRL-2420™) were purchased from ATCC, cultured according to their recommendations and routinely tested for contamination.

### Transplantation procedure

The desired number of cells (ranging from ∼1000 to 6000) were microinjected directly into the blastoderm of 3- to 5-hpf zebrafish blastulae. Transplanted cells were quantified by injecting into PBS and subsequently analyzed by flow cytometry. For a complete transplantation protocol description, see Supplementary Materials and Methods.

### Cell labeling

For labeling and *in vivo* visualization, cells were incubated with the vital dye CellTrace™ Violet Cell Proliferation Kit (450 nm blue emission) (Thermo Fisher Scientific Inc.) or CellTrace™ CSFE (517 nm green emission) (Thermo Fisher Scientific Inc.) according to the manufacturer's recommendations.

### Infection and bacterial strains

A clinical isolate of *K. pneumoniae* was used for infection experiments. For *in vivo* visualization, the bacteria were transformed with the pRSET-tdTomato vector, where tdTomato is constitutively expressed (Shaner et al., 2008). Bacteria were cultured over night at 37°C in LB media with or without carbenicillin (to select for cells carrying pRSET-tdTomato). For infections, embryos were anesthetized with tricaine (Westerfield, 2000), immobilized in 1% low-melting-point agarose (Sigma) and covered with E3 medium, both containing tricaine. Larvae were microinjected directly into the tail muscles or the otic vesicle. For tail-muscle infection quantification, animals with a range of 25-70 murine CHT-resident cells were used. For the otic-vesicle infection quantification, animals with a range of 0 to up to 5 otic-vesicle-resident cells were used. Bacterial inocula were determined by injecting into PBS and later plating into LB agar plates. After infection, the xenotransplanted animals were incubated at 30°C to favor the mammalian cell response*.*

### Whole-mount immunofluorescence

Zebrafish embryos were euthanized with tricaine in E3 medium, fixed in fresh 4% paraformaldehyde in PBS and incubated in methanol (1 h at −20°C). Embryos were rehydrated using decreasing concentrations of methanol in PBST (PBS, 0.3% Triton X-100) and permeabilized using 1 μg/ml proteinase K at room temperature for 1 h. Larvae were then re-fixed and incubated with a cold ethanol/acetone solution (2:1) for 10 min at −20°C. Fixed and permeabilized embryos were then incubated in blocking solution (10 mg/ml BSA, 2% BFS, 1% DMSO, 0.1% Triton X-100) and later incubated either with the anti-mouse c-kit biotin-conjugated antibody (1:100, Abcam, #ab25022), the anti-mouse Ly-6G/Ly-6C (Gr-1) biotin-conjugated antibody (BioLegend, #108404), the anti-mouse Ki-67-FITC (1:100, Ebioscience, #11-5698-80) or the anti-mouse F4/80-FITC antibody (1:100, Ebioscience, #11-4801-82). Streptavidin-FITC (1:500, Ebioscience, #11-4317-87) was used as secondary antibody for biotin-conjugated primary antibodies. For quantification, the CHT of chimeric animals was visualized and manually counted for positive murine cells for a specific antibody on an epifluorescence microscope (Zeiss Axio Observer). Percentage was calculated with respect to the total murine CHT cell count in individual larvae (blue-labeled cells). Mean and s.e.m. was calculated from total larvae analyzed.

### TUNEL assays

Zebrafish embryos were euthanized, fixed, permeabilized and re-fixed as described for whole-mount immunofluorescence. For the terminal deoxynucleotidyl transferase (TdT) dUTP nick-end labeling (TUNEL) assay, the ApopTag^®^ Red In Situ Apoptosis Detection Kit (Millipore Sigma, #S7165) was used. Briefly, fixed and permeabilized embryos were incubated in equilibration buffer for 1 h at room temperature, and then incubated in a reaction mix (20 ml of equilibration buffer, 12 ml of reaction buffer, 6 ml of TdT enzyme, 0.5 ml of 10% Triton X-100) overnight at 37°C. Larvae were then washed several times with PBST and then incubated in blocking solution (10 mg/ml BSA, 2% BFS, 1% DMSO, 0.1% Triton X-100). Anti-digoxigenin-rhodamine (1:50, Roche, #11207750910) was used as conjugated primary antibody. Quantification was performed as described above.

### Time-lapse confocal fluorescence imaging of live zebrafish embryos and larvae

Embryos were anesthetized with tricaine, immobilized in 1% low-melting-point agarose (Sigma) containing tricaine on 35-mm glass-bottom dishes (MatTek), covered with E3 medium containing tricaine and imaged on an epifluorescence microscope (Zeiss Axio Observer), an A1R or C2 (Nikon) confocal microscope, or an Eclipse Ti (Nikon) spinning disk confocal microscope as previously described (Renaud et al., 2011). All images were adjusted for brightness and contrast to improve visualization.

### Flow cytometry analyses

Murine pre- and post-enrichment bone marrow cells were incubated with LIVE/DEAD™ Fixable Near-IR Dead Cell Stain (Thermo Fisher Scientific Inc., #L34975) and the anti-CD45-FITC (1:100, BioLegend, #103107), anti-mouse c-kit-APC (1:100, BioLegend, #105811) and anti-mouse CD11b-PerCP/Cy5.5 (1:100, BioLegend, #101227) primary antibodies. Samples were then analyzed in a NovoCyte flow cytometer (ACEA Biosciences Inc.). For zebrafish chimera cell analyses at 2 dpf, embryos were euthanized with tricaine in E3 medium (8-12 embryos per condition) and mechanically dissociated by sterile razor blades or 0.05% trypsin (Gibco™). A single-cell suspension was prepared by collecting dissociated tissue in 0.5 ml 0.9× PBS/2% FBS and passing the sample through a 40-µM nylon mesh. Samples were subjected to 3 nM DRAQ-7 Dead Cell Stain (Abcam, #ab109202) or LIVE/DEAD™ Fixable Near-IR Dead Cell Stain and incubated with the aforementioned antibodies or Ter119-APC (1:100, BioLegend, #116211), CD19-APC (1:100, BioLegend, #152409), CD3-APC (1:100, BioLegend, #100235), Gr1-APC (1:100, BioLegend, #108411), F4/80-APC (1:100, BioLegend, #123115) or CD11b-PE (1:100, BioLegend, #101207) and analyzed by flow cytometry on a BD FACSAria II (Becton Dickinson) or a NovoCyte flow cytometer. Data analysis was performed in FlowJo v10.

### Statistical analyses

All results were analyzed with the GraphPad Prism 7.0 statistical software and presented as the arithmetic mean±s.e.m. or s.d. Linear correlation, one-way ANOVA or unpaired 2-tailed Student's *t*-tests were performed for statistical analysis as described for each graph in its corresponding figure legend.

## Supplementary Material

Supplementary information

First Person interview

## References

[DMM034876C1] Alvarez M., Reynaert N., Chávez M. N., Aedo G., Araya F., Hopfner U., Fernández J., Allende M. L. and Egaña J. T. (2015). Generation of viable plant-vertebrate chimeras. *PLoS ONE* 10, e0130295 10.1371/journal.pone.0130295 26126202 PMC4488345

[DMM034876C2] Avraham R., Haseley N., Brown D., Penaranda C., Jijon H. B., Trombetta J. J., Satija R., Shalek A. K., Xavier R. J., Regev A. et al. (2015). Pathogen cell-to-cell variability drives heterogeneity in host immune responses. *Cell* 162, 1309-1321. 10.1016/j.cell.2015.08.027 26343579 PMC4578813

[DMM034876C3] Beltman J. B., Marée A. F. M. and de Boer R. J. (2009). Analysing immune cell migration. *Nat. Rev. Immunol.* 9, 789-798. 10.1038/nri2638 19834485

[DMM034876C4] Bertrand J. Y., Kim A. D., Teng S. and Traver D. (2008). CD41+ cmyb+ precursors colonize the zebrafish pronephros by a novel migration route to initiate adult hematopoiesis. *Development* 135, 1853-1862. 10.1242/dev.015297 18417622 PMC2762343

[DMM034876C5] Corkery D. P., Dellaire G. and Berman J. N. (2011). Leukaemia xenotransplantation in zebrafish-chemotherapy response assay *in vivo*. *Br. J. Haematol.* 153, 786-789. 10.1111/j.1365-2141.2011.08661.x 21517816

[DMM034876C6] de Jong J. L. O. and Zon L. I. (2005). Use of the zebrafish system to study primitive and definitive hematopoiesis. *Annu. Rev. Genet.* 39, 481-501. 10.1146/annurev.genet.39.073003.095931 16285869

[DMM034876C7] Ellett F., Pase L., Hayman J. W., Andrianopoulos A. and Lieschke G. J. (2011). mpeg1 promoter transgenes direct macrophage-lineage expression in zebrafish. *Blood* 117, e49-e56. 10.1182/blood-2010-10-314120 21084707 PMC3056479

[DMM034876C8] Fior R., Póvoa V., Mendes R. V., Carvalho T., Gomes A., Figueiredo N. and Ferreira M. G. (2017). Single-cell functional and chemosensitive profiling of combinatorial colorectal therapy in zebrafish xenografts. *Proc. Natl. Acad. Sci. USA*, 114, E8234-E8243. 10.1073/pnas.1618389114 28835536 PMC5625889

[DMM034876C9] Herbomel P., Thisse B. and Thisse C. (1999). Ontogeny and behavior of early macrophages in the zebrafish embryo. *Development* 126, 3735-3745. 10433904 10.1242/dev.126.17.3735

[DMM034876C10] Ito R., Takahashi T., Katano I. and Ito M. (2012). Current advances in humanized mouse models. *Cell. Mol. Immunol.* 9, 208-214. 10.1038/cmi.2012.2 22327211 PMC4012844

[DMM034876C11] Kaushansky A., Mikolajczak S. A., Vignali M. and Kappe S. H. I. (2014). Of men in mice: the success and promise of humanized mouse models for human malaria parasite infections. *Cell. Microbiol.* 16, 602-611. 10.1111/cmi.12277 24506682 PMC4008334

[DMM034876C12] Kikuchi K., Holdway J. E., Major R. J., Blum N., Dahn R. D., Begemann G. and Poss K. D. (2011). Retinoic acid production by endocardium and epicardium is an injury response essential for zebrafish heart regeneration. *Dev. Cell* 20, 397-404. 10.1016/j.devcel.2011.01.010 21397850 PMC3071981

[DMM034876C14] Konantz M., Balci T. B., Hartwig U. F., Dellaire G., André M. C., Berman J. N. and Lengerke C. (2012). Zebrafish xenografts as a tool for in vivo studies on human cancer. *Ann. N. Y. Acad. Sci.* 1266, 124-137. 10.1111/j.1749-6632.2012.06575.x 22901264

[DMM034876C15] Li P., Lahvic J. L., Binder V., Pugach E. K., Riley E. B., Tamplin O. J., Panigrahy D., Bowman T. V., Barrett F. G., Heffner G. C. et al. (2015). Epoxyeicosatrienoic acids enhance embryonic haematopoiesis and adult marrow engraftment. *Nature* 523, 468-471. 10.1038/nature14569 26201599 PMC4754787

[DMM034876C16] Lister J. A., Robertson C. P., Lepage T., Johnson S. L. and Raible D. W. (1999). nacre encodes a zebrafish microphthalmia-related protein that regulates neural-crest-derived pigment cell fate. *Development* 126, 3757-3767. 10433906 10.1242/dev.126.17.3757

[DMM034876C17] Lu J. W., Hsieh M. S., Liao H. A., Yang Y. J., Ho Y. J. and Lin L. I. (2015). Zebrafish as a model for the study of human myeloid malignancies. *Biomed. Res. Int.* 2015, 641475. 26064935 10.1155/2015/641475 PMC4433643

[DMM034876C18] Marques I. J., Weiss F. U., Vlecken D. H., Nitsche C., Bakkers J., Lagendijk A. K., Partecke L. I., Heidecke C.-D., Lerch M. M. and Bagowski C. P. (2009). Metastatic behaviour of primary human tumours in a zebrafish xenotransplantation model. *BMC Cancer* 9, 128 10.1186/1471-2407-9-128 19400945 PMC2697170

[DMM034876C19] Mosimann C., Kaufman C. K., Li P., Pugach E. K., Tamplin O. J. and Zon L. I. (2011). Ubiquitous transgene expression and Cre-based recombination driven by the ubiquitin promoter in zebrafish. *Development* 138, 169-177. 10.1242/dev.059345 21138979 PMC2998170

[DMM034876C20] Munoz-Price L. S., Poirel L., Bonomo R. A., Schwaber M. J., Daikos G. L., Cormican M., Cornaglia G., Garau J., Gniadkowski M., Hayden M. K. et al. (2013). Clinical epidemiology of the global expansion of Klebsiella pneumoniae carbapenemases. *Lancet Infect. Dis.* 13, 785-796. 10.1016/S1473-3099(13)70190-7 23969216 PMC4673667

[DMM034876C21] Murayama E., Kissa K., Zapata A., Mordelet E., Briolat V., Lin H.-F., Handin R. I. and Herbomel P. (2006). Tracing hematopoietic precursor migration to successive hematopoietic organs during zebrafish development. *Immunity* 25, 963-975. 10.1016/j.immuni.2006.10.015 17157041

[DMM034876C22] Paik E. J. and Zon L. I. (2010). Hematopoietic development in the zebrafish. *Int. J. Dev. Biol.* 54, 1127-1137. 10.1387/ijdb.093042ep 20711990

[DMM034876C23] Paul C. D., Devine A., Bishop K., Xu Q., Wulftange W. J., Burr H., Daly K. M., Lewis C., Green D. S., Staunton J. R., et al. (2017). Human macrophages survive and adopt activated genotypes in living zebrafish. *bioRxiv*. 10.1101/181685 PMC6370805 30741975

[DMM034876C24] Reinisch A., Thomas D., Corces M. R., Zhang X., Gratzinger D., Hong W.-J., Schallmoser K., Strunk D. and Majeti R. (2016). A humanized bone marrow ossicle xenotransplantation model enables improved engraftment of healthy and leukemic human hematopoietic cells. *Nat. Med.* 22, 812-821. 10.1038/nm.4103 27213817 PMC5549556

[DMM034876C13] Renaud O., Herbomel P. and Kissa K. (2011). Studying cell behavior in whole zebrafish embryos by confocal live imaging: application to hematopoietic stem cells. *Nat. Protoc.* 6, 1897-1904. 10.1038/nprot.2011.408 22082984

[DMM034876C25] Renshaw S. A. and Trede N. S. (2012). A model 450 million years in the making: zebrafish and vertebrate immunity. *Dis. Model. Mech.* 5, 38-47. 10.1242/dmm.007138 22228790 PMC3255542

[DMM034876C26] Renshaw S. A., Loynes C. A., Trushell D. M. I., Elworthy S., Ingham P. W. and Whyte M. K. B. (2006). A transgenic zebrafish model of neutrophilic inflammation. *Blood* 108, 3976-3978. 10.1182/blood-2006-05-024075 16926288

[DMM034876C27] Robertson A. L., Holmes G. R., Bojarczuk A. N., Burgon J., Loynes C. A., Chimen M., Sawtell A. K., Hamza B., Willson J., Walmsley S. R. et al. (2014). A zebrafish compound screen reveals modulation of neutrophil reverse migration as an anti-inflammatory mechanism. *Sci. Transl. Med.* 6, 225ra29 10.1126/scitranslmed.3007672 PMC4247228 24574340

[DMM034876C28] Sacco A., Roccaro A. M., Ma D., Shi J., Mishima Y., Moschetta M., Chiarini M., Munshi N., Handin R. I. and Ghobrial I. M. (2016). Cancer cell dissemination and homing to the bone marrow in a Zebrafish model. *Cancer Res.* 76, 463-471. 10.1158/0008-5472.CAN-15-1926 26744527

[DMM034876C29] Shaner N. C., Lin M. Z., McKeown M. R., Steinbach P. A., Hazelwood K. L., Davidson M. W. and Tsien R. Y. (2008). Improving the photostability of bright monomeric orange and red fluorescent proteins. *Nat. Methods* 5, 545-551. 10.1038/nmeth.1209 18454154 PMC2853173

[DMM034876C30] Shultz L. D., Brehm M. A., Garcia-Martinez J. V. and Greiner D. L. (2012). Humanized mice for immune system investigation: progress, promise and challenges. *Nat. Rev. Immunol.* 12, 786-798. 10.1038/nri3311 23059428 PMC3749872

[DMM034876C31] Staal F. J. T., Spaink H. P. and Fibbe W. E. (2016). Visualizing human Hematopoietic Stem Cell trafficking *in vivo* using a zebrafish xenograft model. *Stem Cells Dev.* 25, 360-365. 10.1089/scd.2015.0195 26650921

[DMM034876C32] Subramanian N., Torabi-Parizi P., Gottschalk R. A., Germain R. N. and Dutta B. (2015). Network representations of immune system complexity. *Rev. Syst. Biol. Med.* 7, 13-38. 10.1002/wsbm.1288 PMC4339634 25625853

[DMM034876C33] Toh M. C., Goodyear M., Daigneault M., Allen-Vercoe E. and Van Raay T. J. (2013). Colonizing the embryonic zebrafish gut with anaerobic bacteria derived from the human gastrointestinal tract. *Zebrafish* 10, 194-198. 10.1089/zeb.2012.0814 23530761

[DMM034876C34] Torraca V., Masud S., Spaink H. P. and Meijer A. H. (2014). Macrophage-pathogen interactions in infectious diseases: new therapeutic insights from the zebrafish host model. *Dis. Model. Mech.* 7, 785-797. 10.1242/dmm.015594 24973749 PMC4073269

[DMM034876C35] Trede N. S., Langenau D. M., Traver D., Look A. T. and Zon L. I. (2004). The use of zebrafish to understand immunity. *Immunity* 20, 367-379. 10.1016/S1074-7613(04)00084-6 15084267

[DMM034876C36] Westerfield M. (2000). *The Zebrafish Book: A Guide for the Laboratory Use of Zebrafish (Danio rerio)*, 4th edn. University of Oregon Press.

[DMM034876C37] Willett C. E., Zapata A. G., Hopkins N. and Steiner L. A. (1997). Expression of zebrafish rag genes during early development identifies the thymus. *Dev. Biol.* 182, 331-341. 10.1006/dbio.1996.8446 9070331

[DMM034876C38] Zhang B., Shimada Y., Kuroyanagi J., Umemoto N., Nishimura Y. and Tanaka T. (2014). Quantitative phenotyping-based *in vivo* chemical screening in a zebrafish model of leukemia stem cell xenotransplantation. *PLoS ONE* 9, e85439 10.1371/journal.pone.0085439 24454867 PMC3893211

[DMM034876C39] Zon L. I. and Peterson R. T. (2005). *In vivo* drug discovery in the zebrafish. *Nat. Rev. Drug Discov.* 4, 35-44. 10.1038/nrd1606 15688071

