# Supplementary material for: Generation of mouse-zebrafish hematopoietic tissue chimeric embryos for hematopoiesis and host-pathogen interaction studies
Source: Dis Model Mech. 2018 Nov 5;11(11):dmm034876. doi: 10.1242/dmm.034876 (PMC6262816; doi:10.1242/dmm.034876)
Supplement: Supplementary information [file dmm-11-034876-s1.pdf]

© 2018. Published by The Company of Biologists Ltd | Disease Models & Mechanisms (2018): doi:10.1242/dmm.034876: Supplementary information

FIRST PERSON

First person – Margarita Parada-Kusz

First Person is a series of interviews with the first authors of a selection of papers published in Disease Models & Mechanisms, helping early-career researchers promote themselves alongside their papers. Margarita Parada-Kusz is first author on 'Generation of mouse-zebrafish hematopoietic tissue chimeric embryos for hematopoiesis and host-pathogen interaction studies', published in DMM. Margarita is a Research Fellow in the lab of Deborah Hung at Massachusetts General Hospital, Boston, USA. Her main research interest is the study of inflammation using zebrafish as a disease model.

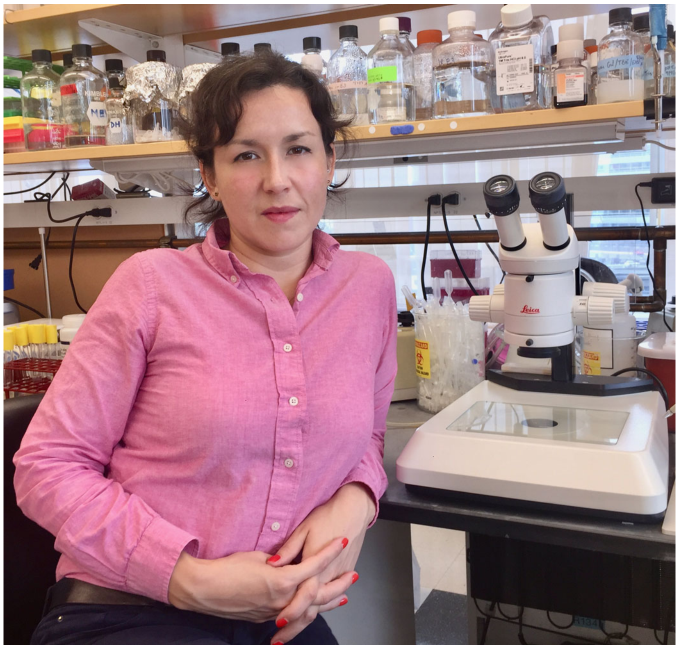

Margarita Parada-Kusz

How would you explain the main findings of your paper to non-scientific family and friends?

Animal disease models are fundamental for studying human diseases and discovering new therapeutic targets. Different animal models have different pros and cons. The utilization of various animal models favors clinical research because a wider variety of aspects related to disease can be questioned. For example, the mouse model is good to study blood cell-related diseases because mouse cells are similar to human ones. However, it is difficult to visualize and follow blood cell migration, which is a fundamental process of blood cells, inside the mouse body. The zebrafish, instead, is ideally suited to these studies because the animals are transparent in the early stages of development. This allows direct visualization of blood cell function and migration throughout the animal's body. We have developed a method to generate zebrafish animals that have integrated mouse blood cells into their own blood system. These animals combine the best of two worlds; they allow direct visualization of mouse blood cell function and migration in living organisms. This is advantageous for the study of blood cell diseases.

> "We have developed a method to generate zebrafish animals that have integrated mouse blood cells into their own blood system."

What are the potential implications of these results for your field of research?

We have provided proof-of-principle that our method allows fast study of diverse mammalian blood cell-related processes *in vivo*. For example, we can observe mouse blood cell homing to hematopoietic tissues and immune cell interactions with pathogenic bacteria. Mouse-zebrafish chimeric animals could be used as an experimental system to conduct drug screens looking for molecules that favor human bone marrow transplantation outcomes or favor host antimicrobial responses. More importantly, we have shown that, with our methodology, zebrafish animals containing human blood cells can also be generated. Prospectively, this method could be used as a platform to conduct targeted drug screens or even patient-specific assays to treat human blood-related diseases, which are labor intensive and more expensive to conduct in other mammalian models.

What are the main advantages and drawbacks of the model system you have used as it relates to the disease you are investigating?

Our model system has two main advantages. First, it allows for the generation of hundreds of animals with mouse or human cells that can be used in a variety of experimental settings. Second, it allows minimally invasive evaluation of blood cell function dynamics and at single-cell resolution. The major drawback is that the presence of exogenous cells in these animals is transient, which limits the time frame for experiments to be conducted.

What has surprised you the most while conducting your research?

While doing these experiments, it was particularly impressive to observe how murine and human blood cells incorporate into the fish blood system and function as if they were inside their own natural host. This is strong biological evolutionary evidence on how highly conserved hematopoiesis is between zebrafish and mammals.

Describe what you think is the most significant challenge impacting your research at this time and how will this be addressed over the next 10 years?

In different individuals, different biological components might be responsible for disease development. Trying to capture individual variation poses a unique challenge to disease model research. The possibility to easily and quickly create animals, such as zebrafish, that harbor patient-specific tissue, would be a major advance in clinical research. The future of medicine relies on the possibility to

Margarita Parada-Kusz's contact details: Massachusetts General Hospital, Boston, MA 02114, USA.
E-mail: parada@molbio.mgh.harvard.edu

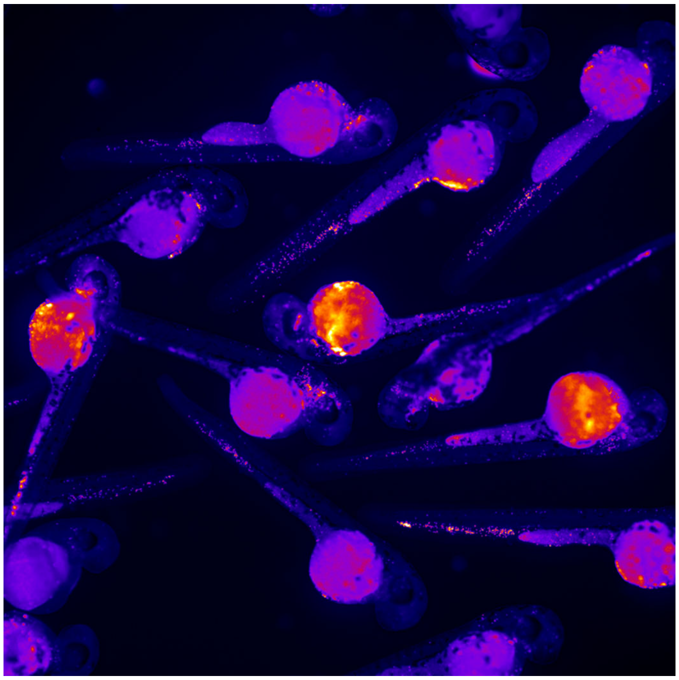

Mouse-zebrafish chimeric animals showing mouse cells (pink) in the hematopoietic tissue. Areas with concentrated mouse cells can be seen orange colored in the fish yolk.

conduct personalized medicine in which personal genetic and physiological aspects of disease could be interrogated.

“Research is a collective endeavor.”

What changes do you think could improve the professional lives of early-career scientists?

One of the most important things that helped my research success and scientific growth as a PhD student was the opportunity to conduct internships. This facilitated the establishment of collaborations, which is a fundamental aspect of modern scientific discovery. Research is a collective endeavor. Encouraging student training abroad by increasing available fellowships would enhance professional development.

What's next for you?

I am particularly interested in the study of inflammation using zebrafish as a disease model. My goal currently is to learn next-generation sequencing technologies to apply them to the study of immune cell responses to bacterial infections.

Reference

Parada-Kusz, M., Penaranda, C., Hagedorn, E. J., Clatworthy, A., Nair, A. V., Henninger, J. E., Ernst, C., Li, B., Riquelme, R., Jijon, H., Villablanca, E. J., Zon, L. I., Hung, D. and Allende, M. L. (2018). Generation of mouse-zebrafish hematopoietic tissue chimeric embryos for hematopoiesis and host-pathogen interaction studies. *Dis. Model. Mech.* **11**, dmm034876.

Disease Models & Mechanisms
